# Transcriptional Comparison Investigating the Influence of the Addition of Unsaturated Fatty Acids on Aroma Compounds During Alcoholic Fermentation

**DOI:** 10.3389/fmicb.2019.01115

**Published:** 2019-05-22

**Authors:** Guo-Liang Yan, Liang-Liang Duan, Pei-Tong Liu, Chang-Qing Duan

**Affiliations:** ^1^Centre for Viticulture and Enology, College of Food Science and Nutritional Engineering, China Agricultural University, Beijing, China; ^2^Key Laboratory of Viticulture and Enology, Ministry of Agriculture, Beijing, China; ^3^College of Public Health, Shaanxi University of Chinese Medicine, Xianyang, China

**Keywords:** unsaturated fatty acids, microarray analyses, *Saccharomyces cerevisiae*, volatile aroma compounds, wine

## Abstract

The levels of unsaturated fatty acids (UFAs) in grape must significantly influence yeast metabolism and the production of aroma compounds. In this work, cDNA microarray technology was applied to analyze the transcriptional discrepancies of wine yeast (commercial wine yeast Lalvin EC1118) fermenting in synthetic grape must supplemented with different concentrations of a mixture of UFAs (including linoleic acid, oleic acid, and α-linolenic acid). The results showed that the initial addition of a high level of UFAs can significantly enrich the intracellular UFAs when compared to a low addition of UFAs and further increase the cell population and most volatiles, including higher alcohols and esters, except for several fatty acids. Microarray analyses identified that 63 genes were upregulated, and 91 genes were downregulated during the different fermentation stages. The up-regulated genes were involved in yeast growth and proliferation, stress responses and amino acid transportation, while the repressed genes were associated with lipid and sterol biosynthesis, amino acid metabolism, TCA cycle regulation, mitochondrial respiration, and stress responses. Unexpectedly, the genes directly related to the biosynthesis of volatile compounds did not vary substantially between the fermentations with the high and low UFA additions. The beneficial aromatic function of the UFAs was ascribed to the increased biomass and amino acid transportation, considering that the incorporation of the additional UFAs in yeast cells maintains high membrane fluidity and increases the ability of the cells to resist deleterious conditions. Our results highlighted the importance of UFAs in the regulation of aroma biosynthesis during wine fermentation and suggested that the improvement of the resistance of yeast to extreme stresses during alcoholic fermentation is essential to effectively modulate and improve the production of aroma compounds. A potential way to achieve this goal could be the rational increase of the UFA contents in grape must.

## Introduction

The production of aroma compounds during wine fermentation is largely influenced by the nutrition status of grape must. Even a small change in the must composition and nutrition concentration, such as sugar concentration, nitrogen source (amino acids), vitamins and fatty acids, could result in a significant impact on the profile of aroma compounds (Bell and Henschke, 2005; Luan et al., 2018). In this context, the effect of assimilable nitrogen sources (YAN) on the formation of volatile compounds has been investigated exhaustively because changes in the YAN content (ammonium salts or amino acids) have a direct and specific effect on the aroma quality of wine (Marks et al., 2003; Mendes-Ferreira et al., 2007). In recent years, the importance of unsaturated fatty acids (UFAs) for yeast fermentation performance and volatile formations has been recognized by winemakers. UFAs are required by *Saccharomyces cerevisiae* to grow under anaerobic conditions. The incorporation of more UFAs into yeast cells can maintain membrane integrity and increase their ability to resist fermentation stresses, such as high sugar and ethanol toxicity (Holcberg and Margalith, 1981; You et al., 2003). In addition, the degree of unsaturation of the cell membrane can influence the activity of membrane-associated enzymes and transporters (such as ATPase and general amino acid permease) and modulate the production of aroma compounds (Calderbank et al., 1984; Rosa and Sa-Correia, 1992). The absence of oxygen during wine fermentation suppresses the fatty acid desaturation of yeast. An alternative to biosynthesis is the uptake of UFAs from grape juice to avoid stuck fermentation (Varela et al., 2012). Several works demonstrated that UFAs influence the production of volatile compounds via their regulation of the formation of precursor acyl-CoA and the expression of related genes (Yoshioka and Hashimoto, 1983; Trotter, 2001; Swiegers et al., 2005; Duan et al., 2015; Rollero et al., 2016). The aromatic functions of UFAs are largely dependent on the type and concentration of UFAs, which might explain why the results reported by different researchers are inconsistent. For example, the addition of oleic acid and ergosterol can increase the production of higher alcohols and acetate esters but inhibit 1-butanol and 1-pentanol (Mauricio et al., 1997). Casu et al. (2016) found that increasing the concentration of linoleic acid was unfavorable for acetate ester formation but improved the production of higher alcohols. Fujii et al. (1997) confirmed that supplementation with linoleic acid can inhibit AATase (alcohol acetyltransferase) activity and reduce acetate ester synthesis. In a study of this synergistic effect, Tween 80, containing 70% oleic acid and 30% palmic acid and stearic acid, was added to improve the content of esters, higher alcohols and volatile fatty acids of wine (Varela et al., 2012).

Grape berries contain 0.15–0.24% (wet weight basis) lipids (Gallander and Peng, 1980), with UFAs being the major components of the total lipids. Linoleic acid (C18:2n6) is the most abundant lipid, followed by oleic (C18:1n9) and α-linolenic acids (C18:3n3). UFA concentrations in grape must change with grape cultivars (Ancín et al., 1998), fermentation technologies such as grape must clarification (Varela et al., 1999) and grape-skin maceration (Valero et al., 1998). Therefore, from the wine production perspective, it is essential to investigate the synergistic effect of UFAs on aroma compound synthesis during wine fermentation. Duan et al. (2015) indicated that rationally increasing the concentrations of UFA mixtures (linoleic, oleic and α-linolenic acids) can improve yeasts growth and most volatile compounds in wine, including higher alcohols, acetate esters (isoamyl acetate and 2-phenylethyl acetate) and ethyl esters. Numerous efforts have been made to characterize the entire gene expression profiles under different nitrogen conditions during vinification (Marks et al., 2003; Mendes-Ferreira et al., 2007; Liu et al., 2018). However, to our knowledge, no related information is available on the response of *S. cerevisiae* to UFA variation during wine fermentation. In this work, cDNA microarray technology was therefore applied to analyze the transcriptional discrepancies of the wine yeast *S. cerevisiae* EC1118 fermenting in two different culture media with different levels of UFAS (including linoleic, oleic, and α-linolenic acids), in which various aromatic compound profiles were detected. To facilitate this investigation, a simplified, chemically defined medium (MS300) that resembles the nutrient composition of grape juice was used, which is often employed in the transcriptional research of wine yeasts (Rossignol et al., 2003; Rossouw et al., 2010).

## Materials and Methods

### Yeast Strain and Culture Media

The commercial *S. cerevisiae* var. *bayanus* strain EC1118 (Lallemand Inc., Blagnac, France) was used in this study. It is used for both red and white winemaking worldwide and is considered a fast and robust fermenting strain (Brice et al., 2014). The nitrogen synthetic grape must MS300 was used in this work (Varela et al., 2004). The pH of the medium was adjusted to 3.3. According to previous data by Duan et al. (2015) two UFA mixture concentrations (including linoleic, oleic and α-linolenic acids purchased from Sigma-Aldrich Company, St. Louis, MO, United States) were added to the MS300 medium, and a high UFA concentration medium (with 390 mg/L linoleic, 130 mg/L oleic, and 104 mg/L α-linolenic acids) and a low UFA concentration medium (with 30 mg/L linoleic, 10 mg/L oleic, and 8 mg/L α-linolenic acids) required to ensure normal cell growth and fermentation, considered the control, were obtained.

### Fermentation Conditions and Samples

*Saccharomyces cerevisiae* EC1118 strain was inoculated into 200 mL YEPD medium (10 g/L yeast extract, 20 g/L peptone, 20 g/L glucose) and cultivated for approximately 15 h with shaking (120 rpm) at 28°C. After harvesting and washing twice with sterile water, 5 mL of yeast cells suspension was added into the flask, and the initial viable population was approximately 10^6^ CFU/mL. Before the inoculation, nitrogen was sparged to eliminate oxygen from the medium. The 500 mL flasks with 350 mL MS300 medium were sealed with a fermentation lock, which guarantees carbon dioxide exhaustion and prevents oxygen from entering the flasks to achieve anaerobic conditions. Fermentations were carried out without shaking at 25°C and in triplicate. The fermentation lasted 170 h. The progress of fermentation was monitored daily by measuring the cell density (OD_600_) and sugar consumption. A total of 25 mL samples were taken from the fermentation flasks with a puncture needle and were immediately centrifuged to collect the cell-free supernatants for the analysis of the main fermentation products and aroma compounds.

### Analytical Methods

General parameters (glucose, ethanol, glycerol, acetic acid, malic acid, lactic acid, and succinic acid) were determined by high-performance liquid chromatography (HPLC, 1200 series, Agilent Technologies, Inc., Palo Alto, CA, United States) as described by Duan et al. (2015). The system was equipped with an HPX-87H Aminex ion-exchange column (300 mm × 7.8 mm, Bio-Rad Laboratories, Hercules, CA, United States) with 5 mM sulfuric acid as the mobile phase. The volatile compounds of the wines were determined by headspace solid-phase microextraction coupled with gas chromatography-mass spectrometry (HS-SPME-GC-MS) as previously described (Zhang et al., 2011; Xu et al., 2015). An Agilent 6890A equipped with a 5975C MS system and an HP-INNOWAX column (60 m × 0.25 mm × 0.25 μm) was used in this system. The aroma compounds were identified by a comparison of the retention indices (RI) of reference standards and mass spectra that matched in the NIST 08 MS database. The quantification was applied with the calibration curves of aroma standards as described by Xu et al. (2015). Analyses were performed in triplicate. Significant differences of metabolites among the treatments were identified using one-way analysis of variance (ANOVA) followed by Duncan’s test (*p* < 0.05) (SPSS 17.0, SPSS Inc., Chicago, IL, United States).

### Microarray Procedures

For the DNA microarray analyses, the RNA of yeasts in mid-exponential (30 h), early-stationary (87 h) and late-stationary growth phases (123 h) were extracted, corresponding to the time points that 19.4, 67.2, and 88.1% sugars were consumed by yeast in high UFA culture, respectively, and 16.7, 57.6, and 89.9% sugars were consumed in low UFA culture, respectively. Three independent cultures were prepared for the biological repeats. Total RNA was isolated using the hot phenol method (Deed et al., 2011) and assessed by agarose gel electrophoresis and a NanoDrop spectrophotometer ND-1000 (NanoDrop products, DE, United States). The RNA samples were subjected to whole-genomic gene expression profiles (CapitalBio Corporation, Beijing, China). After purification, cDNA and biotin-labeled cRNA syntheses, the cRNA samples were hybridized with Yeast Genome 2.0 (Affymetrix GeneChip) and processed as described by the manufacturer (Affymetrix, CA, United States^1^). Pretreatments were applied to eliminate the sample variation, including background rectification and normalization. The pretreated data were analyzed with the RMA algorithm. A significance analysis of microarray (SAM) was applied to identify the genes that were differentially expressed between the high UFA and low UFA cultures. The threshold for significance was set to allow a median of one false positive per analysis for a false discovery rate (FDR) of <0.05%. Genes of the yeast in high UFA culture whose expression levels were greater than twofold or less than 0.5-fold, relative to the yeast in low UFA culture, were considered to be induced or repressed, respectively. These genes were further categorized by biological process using the *Saccharomyces* Genome Database Gene Ontology Slim Mapper tools (*SGD* GO Slim Mapper^2^).

## Results

### Cell Growth, Sugar Consumption, and Major Aroma Compounds in Low and High UFA Cultures

**FIGURE 1:**
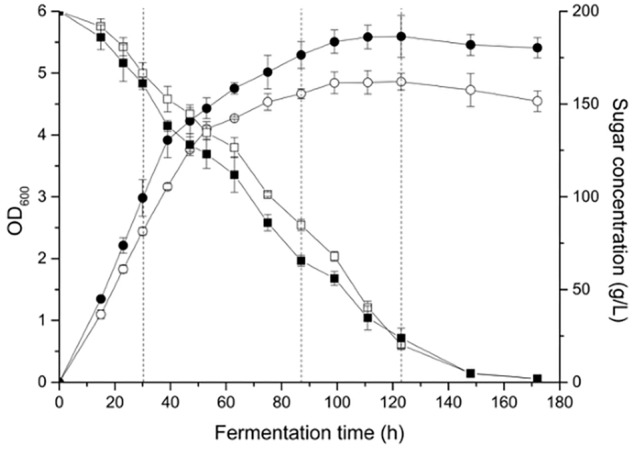
Cell growth and sugar concentration profiles during the fermentation of MS 300 media with high (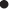, 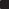) or low (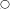, 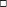) UFA concentrations. Data points represent the mean value from triplicate fermentations, and the vertical bars show ±SD. Dashed lines represent the three growth phases in which the microarray analyses were conducted.

**FIGURE 2:**
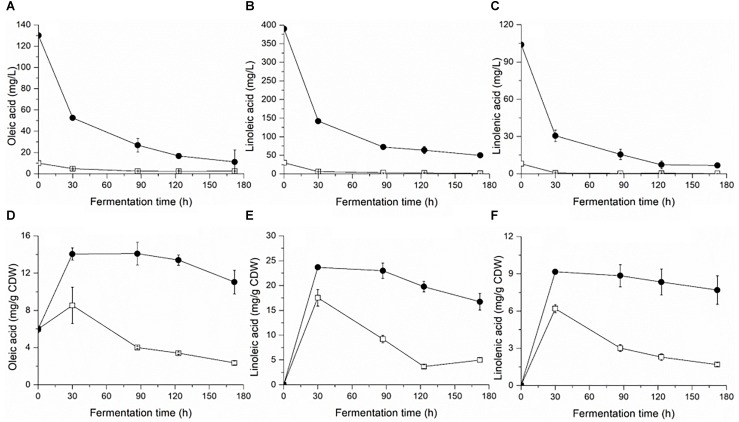
Profiles of extracellular **(A–C)** and intracellular **(D–F)** fatty acids during the fermentation of MS300 media with high (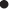) and low (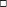) concentrations of UFAs. Data points represent the mean value from triplicate fermentations, and the vertical bars show ±SD.

Yeast cell growth (OD_600_) and sugar consumption in the treatments were monitored during fermentation (Figure 1). To facilitate the comparison, some key kinetic parameters were calculated, such as the duration of fermentation, maximum biomass, time to reach the maximum biomass, maximum specific growth, and rate of fermentation, which are shown in Table 1. In general, a relatively higher level of UFAs improved the yeast population and fermentation activity. The highest cell population (OD_600_) obtained at late-stationary phase (123 h) in the high UFA fermentation (HUF) was 15.0% higher than that in the low UFA fermentation (LUF). Similarly, the fermentation rate (rate of sugar consumption) in HUF was slightly enhanced in comparison to the LUF (2.06 vs. 1.99 g/L⋅h of maximum fermentation rate). The production profiles of ethanol and glycerol were also determined (Supplementary Figure S1). The results showed that the ethanol concentration did not differ between treatments, while glycerol was higher in the HUF (6.34 ± 0.12 vs. 5.93 ± 0.07 g/L in final samples). No significant differences were found in the number of other metabolites, such as acetic, citric and malic acids, in the final samples of both fermentations (data not shown). We further determined the content of extracellular and intracellular UFAs during fermentation (Figure 2). The data indicated that the UFAs added were rapidly taken up by the cells. No UFAs were detected after fermentation in LUF, while few UFAs remained in the HUF sample. As expected, in both treatments, the concentration of intracellular UFAs increased before 30 h fermentation, after which the values slowly decreased. However, the concentration of particular cellular UFAs was always higher in the HUF than in the LUF samples.

**Table 1:** Important parameters of yeast fermentation in MS300 media supplemented with high (HUFA) or low (LUFA) concentrations of UFAs.

Time to reach the end of fermentation (h)		Maximum OD	Time to reach maximum biomass (h)	Maximum specific growth rate (1/h)	Maximum fermentation rate (g/L⋅h)
LUFAs	172	4.86 ± 0.14a	123	0.015 ± 0.01a	1.99
HUFAs	172	5.59 ± 0.34b	123	0.016 ± 0.01a	2.06

**FIGURE 3:**
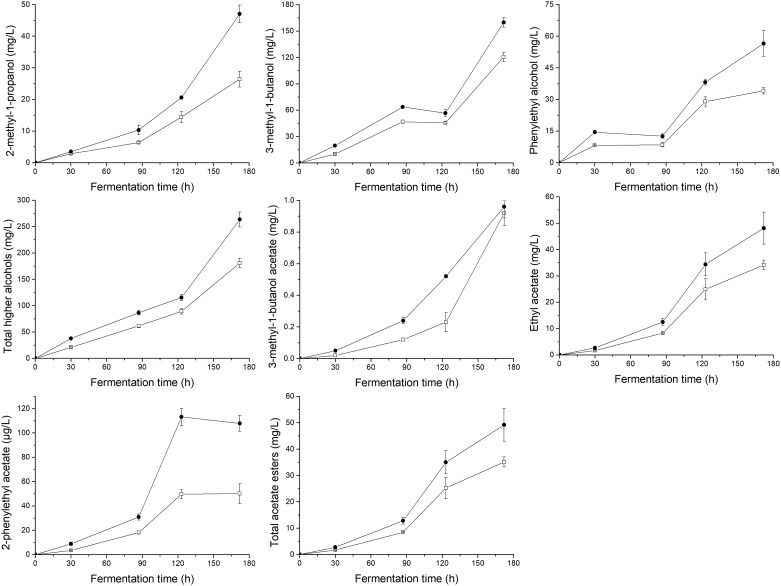
Profiles of the major higher alcohols and total content of higher alcohols, acetate esters and total content of acetate esters during the fermentation of MS300 medium with high (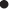) or low (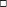) UFA concentrations. Data points represent the mean value from triplicate fermentations, and the vertical bars show ±SD.

To demonstrate the effects of UFAs on the generation of aromatic compounds, the main volatile compounds (higher alcohols, acetate esters, ethyl esters, and fatty acids) produced by yeasts at different fermentation times were determined, as shown in Figure 3, 4. In general, the supply of a relatively higher UFA mixture improved the generation of most aroma compounds, including higher alcohols (2-methyl-1-propanol, 3-methyl-1-butanol, and phenylethyl alcohol) and esters (3-methyl-1-butanol acetate, ethyl acetate, 2-phenylethyl acetate, ethyl hexanoate, ethyl heptanoate, ethyl octanoate, ethyl nonanoate, ethyl decanoate, ethyl dodecanoate, ethyl myristate, and ethyl palmitate). As a result, the total content of higher alcohols, acetate esters and ethyl esters in the final samples of HUF was 45.5, 40.0, and 49.5% higher than those in LUF, respectively. The response of fatty acid formation to the addition of UFA depended to a large extent on the types of fatty acids added. Butanoic acid and octanoic acid showed higher trends, while hexanoic acid, decanoic acid, and dodecanoic acid showed decreased profiles. As a result, no significant difference in total fatty acid content was observed between the two treatments (10.35 ± 0.28 vs. 12.31 ± 0.79 mg/L).

**FIGURE 4:**
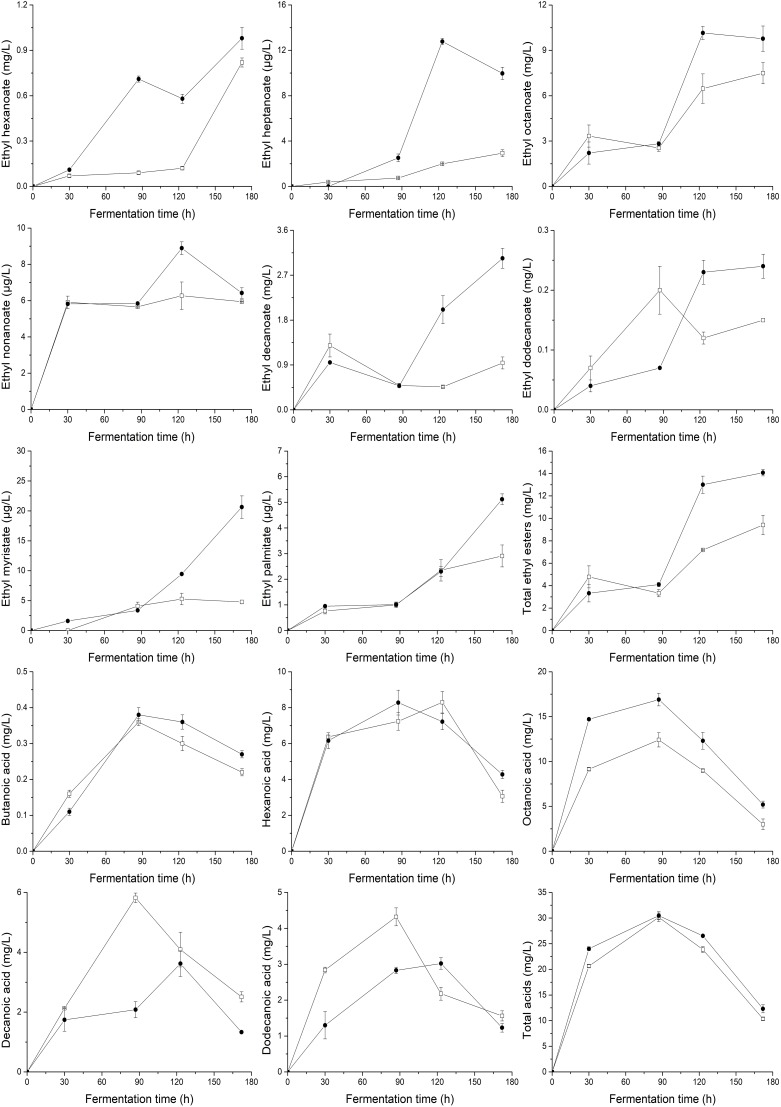
Profiles of major ethyl esters and the total content of ethyl esters, fatty acids and the total content of fatty acids during the fermentation of MS300 media with high (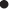) and low (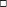) UFA concentrations. Data points represent the mean value from triplicate fermentations, and the vertical bars show ±SD.

### Analysis of Yeast Global Gene Expression by DNA Microarray

Consistent with the results of Duan et al. (2015), the above data indicated that increasing the UFA content in synthetic grape juice improved yeast growth and the production of most volatiles. To gain insight into the mechanism at the molecular level, a comparative transcriptome analysis using DNA microarray technology was applied. The RNA of yeast in the mid-exponential (30 h), early-stationary (87 h), and late-stationary growth phases (123 h) was used for pair wise comparisons of HUF compared to LUF. In total, 63 genes were upregulated (greater than twofold expression), and 91 were down-regulated (less than 0.5-fold expression), as shown in Tables 2, 3. Most of the affected genes were found in the late-stationary growth phase. To confirm the results of the DNA microarray analyses, six genes were randomly selected, including *FAA4*, *BAP3*, *CLB1*, *SPS100*, *ALD3*, and *MGA2*, and qPCR experiments were performed. Correlation analysis showed that the correlation coefficient between the microarray chip and qPCR determinations exceeded 0.887, indicating that the data obtained by DNA microarray are reliable (Supplementary Figure S2).

**Table 2:** Genes upregulated in MS300 media with high vs. low concentrations of UFAs, in different stages of yeast growth categorized by biological process (double or more).

Open reading frame (ORF)	Gene name	Description	Fold change
**Mid-exponential phase**			
**Stress response**			
YCR104W	*PAU3*	Member of the seripauperin multigene family	3.92
YIL176C	*PAU14*	Member of the seripauperin multigene family	2.58
YAL068C	*PAU8*	Member of the seripauperin multigene family	2.90
YDL243C	*AAD4*	Aryl-Alcohol Dehydrogenase	2.62
**Glucose repression**			
YBR066C	*NRG2*	Negative Regulator of Glucose-controlled genes	2.56
**Ergosterol biosynthesis**			
YOR237W	*HES1*	Protein implicated in the regulation of ergosterol biosynthesis	2.78
**Unknown function**			
YML083C	–	Protein of unknown function	2.42
YCR102C	–	Protein of unknown function	2.06
**Early-stationary phase**			
**Sugar utilization**			
YJL219W	*HXT9*	Putative hexose transporter that is nearly identical to Hxt11p	3.47
YJL216C	*IMA5*	Alpha-glucosidase	2.78
**Unknown function**			
YMR317W	–	Putative protein of unknown function	2.58
YKL068W-A	–	Putative protein of unknown function	2.19
Late-stationary phase			
**Cell wall biosynthesis**			
YHR143W	*DSE2*	Daughter cell-specific secreted protein with similarity to glucanases	4.59
YER124C	*DSE1*	Daughter cell-specific protein	2.08
YNR067C	*DSE4*	Daughter cell-specific secreted protein	3.55
YNL327W	*EGT2*	Glycosylphosphatidylinositol (GPI)-anchored cell wall endoglucanase	3.33
YGL028C	*SCW11*	Cell wall protein with similarity to glucanases	3.05
YNL066W	*SUN4*	Cell wall protein related to glucanases	3.77
YHR126C	*ANS1*	Putative GPI protein	2.18
YHR139C	*SPS100*	Protein required for spore wall maturation	2.12
**Cell cycle, RNA, and ribosome**			
YLR286C	*CTS1*	Endochitinase	2.97
YIL158W	*AIM20*	Altered inheritance rate of mitochondria	3.02
YGR108W	*CLB1*	B-type cyclin involved in cell cycle progression	2.29
YDR146C	*SWI5*	Transcription factor that recruits the mediator and Swi/Snf complexes	2.22
YPR119W	*CLB2*	B-type cyclin involved in cell cycle progression	2.02
YGL029W	*CGR1*	Protein involved in nucleolar integrity and processing of pre-rRNA	2.39
YIL016W	*SNL1*	Ribosome-associated protein	2.24
**Metal and ions** **homeostasis**			
YOR079C	*ATX2*	Golgi membrane protein involved in manganese homeostasis	2.23
YLR034C	*SMF3*	Putative divalent metal ion transporter involved in iron homeostasis	2.28
YNL259C	*ATX1*	Cytosolic copper metallochaperone	2.17
**Stress response**			
YLR461W	*PAU4*	Member of the seripauperin multigene family	3.86
YCR104W	*PAU3*	Member of the seripauperin multigene family	3.81
YIL176C	*PAU14*	Member of the seripauperin multigene family	2.48
YLL064C	*PAU18*	Member of the seripauperin multigene family	2.46
YAL068C	*PAU8*	Member of the seripauperin multigene family	3.73
**Amino acid transportation**			
YHL036W	*MUP3*	Low affinity methionine permease, similar to Mup1p	2.31
YDR046C	*BAP3*	Branched-chain amino acid permease	2.29
YNL217W	*PPN2*	Putative serine/threonine-protein phosphatase	2.11
YOR115C	*TRS33*	Core component of transport protein particle (TRAPP) complexes I–III	2.02
YMR169C	*ALD3*	Cytoplasmic aldehyde dehydrogenase	2.04
**Other process**			
YOL155C	*HPF1*	Haze-protective mannoprotein	2.18
YLR084C	*RAX2*	N-glycosylated protein	2.18
YDR033W	*MRH1*	Membrane protein Related to Hsp30p	2.06
YJL219W	*HXT9*	Putative hexose transporter that is nearly identical to Hxt11p	2.74
YBR092C	*PHO3*	Constitutively expressed acid phosphatase similar to Pho5p	2.08
YGR131W	*FHN1*	Functional Homolog of Nce102	2.01
YBR161W	*CSH1*	Mannosylinositol phosphorylceramide (MIPC) synthase catalytic subunit	2.05
YDL059C	*RAD59*	Protein involved DNA double-strand break repair	2.09
YDR139C	*RUB1*	Ubiquitin-like protein with similarity to mammalian NEDD8	2.12
**Unknown function**			
YLR346C	*CIS1*	Putative protein of unknown function found in mitochondria	2.55
YNL046W	–	Putative protein of unknown function	2.04
YNL058C	–	Putative protein of unknown function	2.03
YMR317W	–	Putative protein of unknown function	2.38
YNL277W-A	–	Putative protein of unknown function	2.32
YNR034W-A	–	Putative protein of unknown function	2.31
YDL085C-A	–	Putative protein of unknown function	2.14
YDR524W-A	–	Putative protein of unknown function	2.11
YML007C-A	–	Putative protein of unknown function	2.10
YOL014W	–	Putative protein of unknown function	2.08
YLR285C-A	–	Putative protein of unknown function	2.53
YMR030W-A	–	Putative protein of unknown function	2.50
YML018C	–	Protein of unknown function	2.01
YIL169C	–	Putative protein of unknown function	2.27

**Table 3:** Genes downregulated in MS300 media with high vs. low concentrations of UFAs, in different stages of yeast growth categorized by biological process (double or more).

Open reading frame (ORF)	Gene name	Description	Fold change
**Mid-exponential phase**			
**Glucose transportation**		Hexose transporter with moderate affinity for glucose	
YHR096C	*HXT5*		0.45
**Zinc ion homeostasis**			
YOL101C	*IZH4*	Membrane protein involved in zinc ion homeostasis	0.28
**Other process**			
YBR067C	*TIP1*	Major cell wall mannoprotein with possible lipase activity	0.47
YDL223C	*HBT1*	Shmoo tip protein, substrate of Hub1p ubiquitin-like protein	0.47
**Early-stationary phase**			
**Lipid and sterol biosynthesis**			
YIR033W	*MGA2*	ER membrane protein involved in regulation of *OLE1* transcription	0.47
YMR246W	*FAA4*	Long chain fatty acyl-CoA synthetase	0.41
**Zinc ion homeostasis**			
YOL101C	*IZH4*	Membrane protein involved in zinc ion homeostasis	0.10
YOL002C	*IZH2*	Plasma membrane proteins thought to affect zinc homeostasis	0.43
**Other process**			
YMR175W	*SIP18*	Phospholipid-binding hydrophilin	0.39
YHR033W	–	Putative glutamate 5-kinase	0.48
YPR192W	*AQY1*	Spore-specific water channel	0.45
YBR067C	*TIP1*	Major cell wall mannoprotein with possible lipase activity	0.44
YLR413W	*INA1*	Putative protein of unknown function	0.24
**Late-stationary phase**			
**Lipid and sterol biosynthesis**			
YIR033W	*MGA2*	ER membrane protein involved in regulation of *OLE1* transcription	0.47
YKL187C	*FAT3*	Protein required for fatty acid uptake	0.40
YMR246W	*FAA4*	Long chain fatty acyl-CoA synthetase	0.39
YOR084W	*LPX1*	Peroxisomal matrix-localized lipase	0.49
YGR249W	*MGA1*	Protein similar to heat shock transcription factor	0.47
YDL222C	*FMP45*	Integral membrane protein localized to mitochondria	0.29
YGL162W	*SUT1*	Transcription factor of the Zn(II)2Cys6 family	0.50
YCR091W	*KIN82*	Putative serine/threonine protein kinase	0.48
**Amino acid metabolism**			
YBR132C	*AGP2*	Plasma membrane regulator of polyamine and carnitine transport	0.50
YMR136W	*GAT2*	Protein containing GATA family zinc finger motifs	0.48
YOR348C	*PUT4*	Proline permease	0.43
YMR042W	*ARG80*	Transcription factor involved in regulating arginine-responsive genes	0.41
YPL111W	*CAR1*	Arginase	0.41
**TCA cycle, mitochondrial respiratory**			
YLL041C	*SDH2*	Succinate dehydrogenase	0.49
YDR216W	*ADR1*	Alcohol dehydrogenase regulator	0.46
YML120C	*NDI1*	NADH: ubiquinone oxidoreductase	0.39
YDL085W	*NDE2*	Mitochondrial external NADH dehydrogenase	0.41
YMR303C	*ADH2*	Glucose-repressible alcohol dehydrogenase II	0.38
YLR393W	*ATP10*	Assembly factor for the F0 sector of mitochondrial F1F0 ATP synthase	0.45
YLL018C-A	*COX19*	Protein required for cytochrome c oxidase assembly	0.42
**Zinc ion homeostasis**			
YDR492W	*IZH1*	Membrane protein involved in zinc ion homeostasis	0.46
YOL101C	*IZH4*	Membrane protein involved in zinc ion homeostasis	0.28
**Stress response**			
YMR276W	*DSK2*	Nuclear-enriched ubiquitin-like polyubiquitin-binding protein	0.49
YMR280C	*CAT8*	Zinc cluster transcriptional activator	0.47
YOR028C	*CIN5*	Basic leucine zipper (bZIP) transcription factor of the yAP-1 family	0.44
YMR070W	*MOT3*	Transcriptional repressor and activator with two C2-H2 zinc fingers	0.46
YER143W	*DDI1*	DNA-damage inducible 1 homolog 1 (*S. cerevisiae*)	0.48
YPL190C	*NAB3*	RNA-binding protein, subunit of Nrd1 complex (Nrd1p-Nab3p-Sen1p)	0.48
YOR178C	*GAC1*	Regulatory subunit for Glc7p type-1 protein phosphatase (PP1)	0.48
YLR116W	*MSL5*	Component of commitment complex	0.47
YAR073W	*IMD2*	Inosine monophosphate dehydrogenase	0.47
YMR164C	*MSS11*	Transcription factor	0.46
YPR065W	*ROX1*	Heme-dependent repressor of hypoxic genes	0.41
YGR088W	*CTT1*	Cytosolic catalase T	0.40
YPL230W	*USV1*	Putative transcription factor containing a C2H2 zinc finger	0.38
YBL066C	*SEF1*	Putative transcription factor; has homolog in *Kluyveromyces lactis*	0.37
YIL101C	*XBP1*	Transcriptional repressor	0.36
YHR205W	*SCH9*	AGC family protein kinase	0.36
YOR140W	*SFL1*	Transcriptional repressor and activator	0.30
YER064C	*VHR2*	Null mutation has global effects on transcription	0.45
YDR169C	*STB3*	Ribosomal RNA processing element (RRPE)-binding protein	0.43
YLL010C	*PSR1*	Plasma membrane associated protein phosphatase	0.44
**Other process**			
YLR315W	*NKP2*	Central kinetochore protein and subunit of the Ctf19 complex	0.50
YEL070W	*DSF1*	Putative mannitol dehydrogenase	0.48
YNL307C	*MCK1*	Meiotic and centromere regulatory ser, tyr-Kinase	0.50
YMR104C	*YPK2*	Protein kinase similar to serine/threonine protein kinase Ypk1p	0.47
YBR067C	*TIP1*	Major cell wall mannoprotein with possible lipase activity	0.46
YLR094C	*GIS3*	GIg1-2 suppressor	0.50
YLR446W	–	Putative hexokinase	0.43
YLL013C	*PUF3*	Protein of the mitochondrial outer surface	0.50
YGL169W	*SUA5*	Protein involved in threonylcarbamoyl adenosine biosynthesis	0.49
YPL119C	*DBP1*	Putative ATP-dependent RNA helicase of DEAD-box protein family	0.49
YAL039C	*CYC3*	Cytochrome c heme lyase	0.48
YHR199C-A	*NBL1*	Subunit of the conserved chromosomal passenger complex (CPC)	0.47
YDL223C	*HBT1*	Shmoo tip protein, substrate of Hub1p ubiquitin-like protein	0.39
YBR212W	*NGR1*	RNA binding protein that negatively regulates growth rate	0.36
YHR033W	–	Putative glutamate 5-kinase	0.33
YJR094C	*IME1*	Master regulator of meiosis that is active only during meiotic events	0.44
YGR068C	*ART5*	ADP-ribosyltransferase 5	0.50
**Unknown function**			
YLR267W	*BOP2*	Protein of unknown function	0.48
YPL054W	*LEE1*	Zinc-finger protein of unknown function	0.39
YBL081W	–	Non-essential protein of unknown function	0.48
YDR505C	*PSP1*	Asn and gln rich protein of unknown function	0.44
YPR153W	–	Putative protein of unknown function	0.47
YHR131C	–	Putative protein of unknown function	0.46
YMR291W	*TDA1*	Protein kinase of unknown cellular role	0.45
YNL269W	*BSC4*	Protein of unknown function	0.45
YMR147W	–	Putative protein of unknown function	0.45
YHR105W	*YPT35*	Endosomal protein of unknown function	0.44
YOL084W	*PHM7*	Protein of unknown function	0.44
YGL056C	*SDS23*	Protein involved in cell separation during budding	0.45
YJR115W	–	Putative protein of unknown function	0.43
YDL129W	–	Protein of unknown function	0.40
YLR413W	*INA1*	Putative protein of unknown function	0.40
YNR014W	–	Putative protein of unknown function	0.40
YDL037C	*BSC1*	Protein of unconfirmed function	0.39
YMR206W	–	Putative protein of unknown function	0.27
YGR067C	–	Putative protein of unknown function	0.37

The affected genes were further categorized according to their biological and functional processes as assigned by the *Saccharomyces* Genome Database (SGD). Eight genes were upregulated, and four genes were downregulated in the mid-exponential growth phase. In addition to two unknown function genes (*YML083C* and *YCR102C*), the upregulated genes included *PAU3*, *PAU14*, *PAU8*, and *AAD4*, which are involved in the processes of stress response. In addition, *AAD4* encodes a putative aryl-alcohol dehydrogenase and is involved in the oxidative stress response. *NRG2* mediates glucose repression, and *HES1* is associated with ergosterol biosynthesis. The downregulated genes included *TIP1* (involved in wall protein synthesis), *HXT5* (encodes hexose transporter), *IZH4* (involved in zinc ion homeostasis), and *HBT1* (encodes shmoo tip protein, the substrate of Hub1p ubiquitin-like protein).

Fewer genes were induced in the early-stationary phase compared to the mid-exponential phase, including *HXT9* and *IMA5*, and two unknown function genes (*YMR317W* and *YKL068W-A*). *HXT9* and *IMA5* are involved in sugar transportation and utilization. The expression of genes involved in lipid and fatty acid biosynthesis was downregulated in HUF. Exogenous fatty acids can strongly repress the *de novo* synthesis of lipids and fatty acids in yeasts, as has been reported by Chirala (1992). Although we did not observe the repression of *OLE1* transcription, which encodes delta (9) fatty acid desaturase and is involved in the formation of UFAs, two other genes, *MGA2* (encoding an ER membrane protein that regulates *OLE1* transcription) and *FAA4* (encoding the long chain fatty acyl-CoA synthetase responsible for the importation of long-chain fatty acids), were found to be downregulated. *IZH2* and *IZH4* were continuously repressed in HUF (still depressed in the late-stationary phase); both genes play an important role in zinc metabolism and homeostasis and exhibit elevated expression in zinc-deficient cells. It has been confirmed that their expression is lipid- and oxygen-dependent and linked with sterol metabolism (Lyons et al., 2004). The down-regulation of these genes might be due to the incorporation of more UFAs into the cell membrane, which disturbs *de novo* lipid syntheses. The decreased expression of *TIP1* was observed in this and the last yeast growth stage of HUF.

Most genes were affected after cells entered the late-stationary phase (51 and 78 genes were upregulated and downregulated, respectively). This could be because the nutritional status at this stage became sterile compared to other stages (Backhus et al., 2001; Marks et al., 2003). The induced genes in this stage were mainly associated with cell wall formation, cell cycle, RNA and ribosome biosynthesis, metal and ion metabolism, stress response, amino acid metabolism, process, and unknown function. The repressed genes were related to lipid, sterol, amino acid, carbohydrate metabolism, stress response, zinc ion homeostasis, process, and unknown function. Cell wall formation, cell cycle, RNA and ribosome biosynthesis are associated with cell growth and proliferation. The high expression of the genes *DSE2*, *DSE1*, *DSE4*, *EGT2*, *SCW1*, *SUN4*, *ANS1*, *SPS100*, *CTS1*, *AIM20*, *CLB1*, *SWI5*, *CLB2*, *CGR1*, and *SNL1* was correlated with an increase in the cell population in HUF. Members of *PAU* (*PAU3*, *PAU14*, *PAU14*, *PAU8*, and *PAU18*) and the genes *MUP3*, *BAP3*, and *TRS33*, all involved in amino acid transportation, were induced at this stage in HUF. In addition, *MUP3* encodes a low affinity methionine permease, and *BAP3* encodes a specific branched-chain amino acid permease. Another upregulated gene was *ALD3*, which encodes cytoplasmic aldehyde dehydrogenase and plays a critical role in the conversion of acetaldehyde to acetyl-CoA during growth on non-fermentable carbon sources, which can be induced in response to ethanol stress (Navarro-Aviño et al., 1999). The upregulation of *ALD3* might enable cells to generate more acetyl-CoA for producing esters in HUF. *PHO3* (encodes acid phosphatase to hydrolyze thiamine phosphates) was also upregulated by high UFA addition.

Compared to upregulated genes, more genes were repressed in the late-stationary phase. In addition to *MGA2* and *FAA4* (which were downregulated in the early-stationary phase), the genes *FAT3*, *LPX1*, *FMP45*, *SUT1*, and *MGA1* involved in lipid and sterol metabolism were repressed. *FAT3* encodes the transporter protein Fat3p and is responsible for fatty acid uptake; *SUT1* encodes the Zn(II)2Cys6 family transcription factor and positively regulates sterol uptake genes under anaerobic conditions; *FMP45* encodes an integral membrane protein located in the mitochondria and is required for sporulation and the maintenance of sphingolipid content. These data suggest that *de novo* synthesis and the metabolism of fatty acids and sterol in cells in the HUF were strongly negatively influenced in the late stationary phase. Additionally, several genes involved in amino acid and nitrogen metabolism were downregulated. It should be noted that these amino acids were not different from those present in the group of upregulated genes. They mainly included *AGP2*, *GAT2*, *PUT4*, *ARG80*, and *CAR1*. *AGP2* encodes the plasma membrane regulator of polyamine and carnitine transport and can act as a sensor that transduces environmental signals. *GAT2* encodes the protein containing the GATA family zinc finger motifs and is repressed by leucine. *PUT4* encodes a proline permease with high affinity proline transport. *ARG80* encodes a transcription factor and is involved in the regulation of arginine-responsive genes. *CAR1* encodes arginase that catabolizes arginine to ornithine and urea and controls the formations of ethyl carbamate (EC). Interestingly, some genes associated with the TCA cycle and mitochondrial respiratory chains were repressed, including *SDH2*, *ADR1*, *NDI1*, *NDE2*, and *ADH2*.

It should be noted that a majority of the stress-response genes were upregulated in LUF compared to HUF at this stage, including *DSK2*, *CAT8* (respond to DNA replication stress), *CIN5* (mediates pleiotropic drug resistance and salt tolerance), *MOT3* (transcriptional repressor, activator, cellular adjustment to osmotic stress), *MSS11* (a transcription factor controlling the activation of *FLO11* and *STA2* in response to nutritional signals), *ROX1* (involved in the hyperosmotic stress resistance), *CTT1* (protects from oxidative damage), *USV1* (responds to salt stress and cell wall biosynthesis), *XBP1* (transcriptional repressor, induced by stress or starvation during mitosis), and *PSR1* (plasma membrane associated protein phosphatase and involved in general stress response). These data suggest that yeast cells in LUF might be subjected to more stresses at this stage than those in HUF.

## Discussion

It is well known that wine yeast is challenged by simultaneous and sequential stresses during alcoholic fermentation, especially ethanol toxicity. The cellular membrane is the cell structure most affected by ethanol, which causes an increase in its permeability and leads to unfavorable effects, such as the inhibition of sugar, ammonium and amino acid uptake (Diniz et al., 2017). To maintain membrane stability, *S. cerevisiae* increases the synthesis of UFAs and enriches the plasma membrane UFA content. Alternatively, in the presence of exogenous UFAs, yeast cells can absorb UFAs into the cell membrane directly and improve their resistance to inhospitable environments, which can lead to increased biomass and fermentative activity and consequently modify the production of aroma compounds (Martin et al., 2007; Duan et al., 2015). To better understand the positive aromatic function of UFAs during wine fermentation, in this work, the transcriptional profiles of wine yeast in response to low and high UFA mixture additions were investigated by DNA microarray analyses in synthetic grape medium. The data described indicate that the initial supplementation of a high UFA mixture can promote cell growth and the production of most aromatic compounds, including higher alcohols, acetate esters and ethyl esters, with the exception of several fatty acids (hexanoic acid, decanoic acid, and dodecanoic acid). Microarray analyses identified that sixty-three and ninety-one genes were upregulated (greater than twofold expression) or downregulated (less than 0.5-fold expression), respectively. Because the aim of the study is to reveal the molecular effect of UFAs on aroma compounds, we focused on the groups of genes associated with volatile production in the discussion below, especially the upregulated genes.

The improvement of cell wall formation can increase the resistance of yeast to environmental stresses (Brennan et al., 2013). The expression of genes involved in cell wall formation, cell cycle, RNA, and ribosome biosynthesis was elevated in HUF, which corresponded to an increase in the biomass in HUF. Increasing the cell population in wine fermentation directly promotes the formation of aroma compounds (Varela et al., 2004; Duan et al., 2015). Thus, the beneficial effect of UFAs on aroma compounds observed in this work could be partially ascribed to an increased cell population able to resist deleterious conditions during wine fermentation. *PAU* genes comprise the largest multiple gene family in *S. cerevisiae*, with 24 members, which are induced by different stresses, such as low temperature, low oxygen and wine fermentation conditions (Luo and van Vuuren, 2009). In this study, *PAU3*, *PAU14*, *PAU8*, and *PAU18* were induced at different stages in HUF, which is consistent with the data of Rossignol et al. (2003), who found that 15 PAU/TIR genes were strongly upregulated during wine alcoholic fermentation. Wilcox et al. (2002) suggested that the function of PAU protein involves sterol transport, which might explain the substantial induction of *PAU* expression in HUF. Increased biomass can consume more nutrients (such as sterol, ions, copper, and thiamine) and cause nutrition deficiency, which can induce the expression of related functional genes to replenish these compounds (Liu et al., 2018). This could account for the increased expression of several genes (*PHO3*, *ATX1*, and *ATX2*) by high UFA additions. *PHO3*, which encodes an acid phosphatase, can hydrolyze thiamine phosphates in the periplasmic space and increase cellular thiamine uptake. Thiamine pyrophosphate is a cofactor essential for the activity of pyruvate decarboxylase, and its depletion has a negative effect on yeast carbon metabolism (Hohmann and Meacock, 1998). Increasing the yeast biomass can consume a large amount of thiamine pyrophosphate, resulting in its deficiency in the must (Liu et al., 2018). The upregulation of *ATX1* and *ATX2*, which encode the Mn^2+^ transporter, implies that the metal could also be limited in the cells (Lin and Culotta, 1996).

Amino acid metabolism is of particular interest from a winemaking perspective, as amino acids serve as the precursors of important volatile aroma compounds (Rossouw et al., 2010). In this study, the expression levels of genes directly related to the formation of aroma compounds, such as *BAT1*, *PDC1*, *ATF1*, *EEB1*, *EHT1*, and *IAH1*, were not significantly different between the two treatments, while the genes encoding amino acid permeases were greatly induced in HUF, for example, *BAP3* that encodes a one branched-chain amino acid permease. Branched-chain amino acids (including L-valine, L-leucine, and L-isoleucine) are important flavor precursors in grape must. The elevated expression of *BAP2* and *BAP3* can enable yeast to transport more extracellular amino acids into the cells to produce higher alcohols and corresponding esters (Hazelwood et al., 2008; Trinh et al., 2010). Thus, the upregulation of *BAP3* might be another reason that an increased number of high alcohol and esters were produced in HUF. It should be mentioned that *BAP3* is subject to nitrogen catabolite repression (NCR) and is strongly repressed by yeast-preferred nitrogen (such as ammonium and glutamine) but is depressed when the cells are starved for nitrogen (McCusker and Haber, 1990; Magasanik and Kaiser, 2002). The induction of *BAP3* in the late-stationary growth phase of HUF could be due to the increased biomass, which causes the nitrogen available for yeast to be deficient in comparison with LUF. The incorporation of abundant UFAs into the cell membrane can help the cells maintain normal membrane fluidity and protect the activity of membrane-associated enzymes and transporters, which might, at least partially, lead to the increased production of aroma compounds. Interestingly, we found that *CAR1* and *ARG80*, which are positively involved in ethyl carbamate (EC) formation, were downregulated in HUF. In grape musts, the catabolism of arginine by wine yeasts can produce ornithine and urea. The secreted urea spontaneously reacts with ethanol to generate EC, which causes different cancers in a variety of test animals (Beland et al., 2005). The disruption of *CAR1* can decrease the production of the carcinogen EC during wine fermentation (Schehl et al., 2007). As a result, it is believed that increasing the UFA content in grape must or enriching UFAs in the cell membrane might be a potential way to reduce the formation of EC in wines. This hypothesis merits further study.

The *de novo* synthesis of lipids and fatty acids is repressed in the presence of exogenous fatty acids (Chirala, 1992), and the absorption of UFAs from the environment can inhibit the biosynthesis of UFAs and FA in yeasts (McDonough et al., 1992). The reduction of medium-chain fatty acids (MCFAs) by exogenous UFAs has been reported by Redon et al. (2011). Similarly, we found that MCFA formation and several genes involved in fatty acid transportation and synthesis were repressed in HUF. It was also found that some genes associated with the TCA cycle and mitochondrial respiration (*SDH2*, *ADR1*, *NDI1*, *NDE2*, and *ADH2*) were induced in LUF in comparison with HUF. Currently, we cannot explain these data well. However, the data of Backhus et al. (2001) showed that the wine yeast response to low nitrogen in the late time point of wine fermentation is to switch from a fermentative mode of metabolism to respiration characteristic with a general relief of TCA and respiration genes from glucose repression, which was accompanied by the increased expression of *ADH2*.

It is important to highlight that some results obtained in this study are not consistent with previous data. For example, the supplementation of single UFAs, such as linoleic acid, oleic acid or α-linolenic acid, can inhibit AATase (alcohol acetyltransferase) activity and reduce acetate ester synthesis (Yoshioka and Hashimoto, 1983; Fujii et al., 1997). The inconsistency might be due to differences in the culture medium or (and) the added UFA compositions (single or combined addition) and concentrations. Additionally, many genes with unknown functions were upregulated (14) or downregulated (19) in HUF vs. LUF. For example, *YLR346C* is regulated by transcription factors involved in pleiotropic drug resistance, Pdr1p and Yrr1p (Le Crom et al., 2002). The expression of *YNR034W-A* is regulated by *Msn2*/*Msn4* (Lai et al., 2005). *Msn2* and *Msn4*, which encode stress-responsive transcriptional activators, are activated in response to various stress conditions. These observations highlight the limitation of our understanding of the molecular mechanisms involved in wine yeast survival and metabolism during wine fermentation. Revealing the functions of these genes could help us to rationally control the process of wine fermentation and effectively modulate the formation of aroma compounds.

## Conclusion

The results of the present work indicate that adding high contents of an UFA mixture into synthetic grape medium increased cell growth and the production of most yeast-derived volatile compounds compared to the low UFA-added culture, including higher alcohols and the corresponding esters, with the exception of several fatty acids. Sixty three and ninety one genes were identified by microarray analyses to be upregulated or downregulated, respectively, during alcoholic fermentation. Most of the upregulated genes were involved in yeast growth and proliferation, stress response, and nitrogen compound transportation. There were no genes directly involved in the formation of higher alcohols, and esters were found to be significantly upregulated in HUF vs. LUF. The improvement of aroma compounds in HUF is ascribed to the increased resistance of yeast to various stresses due to the incorporation of more UFAs into cells and the increased biomass and amino acid transportation. Our results highlighted the importance of UFAs in the regulation of aroma biosynthesis during wine fermentation and suggested that improving the resistance of yeast to extreme stresses is essential to effectively manipulate and improve the production of aroma compounds.

## Author Contributions

C-QD and G-LY designed the experiments. L-LD and P-TL conducted the experiments. L-LD, P-TL, and G-LY analyzed the experimental data and wrote the manuscript.

## Conflict of Interest Statement

The authors declare that the research was conducted in the absence of any commercial or financial relationships that could be construed as a potential conflict of interest.

## Footnotes

1 www.affymetrix.com

2 https://yeastmine.yeastgenome.org/yeastmine/tools.do
