# Supplementary material for: Transcriptional Comparison Investigating the Influence of the Addition of Unsaturated Fatty Acids on Aroma Compounds During Alcoholic Fermentation
Source: Front Microbiol. 2019 May 22;10:1115. doi: 10.3389/fmicb.2019.01115 (PMC6538801; doi:10.3389/fmicb.2019.01115)
Supplement: Supplementary file 1 [file Table_1.docx]

Supplementary Material

Transcriptional comparison investigating the influence of the addition of unsaturated fatty acids on aroma compounds during alcoholic fermentation

Guo-Liang Yan^1,2^, Liang-Liang Duan^3^, Pei-Tong Liu^1,2^, Chang-Qing Duan^1,2^*****

* *Author for correspondence (Tel: +86-10-62737136; Fax: +86-10-62738658; E-mail:* [*duanchq@vip.sina.com*](mailto:duanchq@vip.sina.com)*)*

# Supplementary Figures





**Figure S1** Profiles of ethanol and glycerol during the fermentation of MS300 media with high (●) and low (□) concentrations of UFAs. Data points represent the mean value from triplicate fermentations and the vertical bars show ± SD.


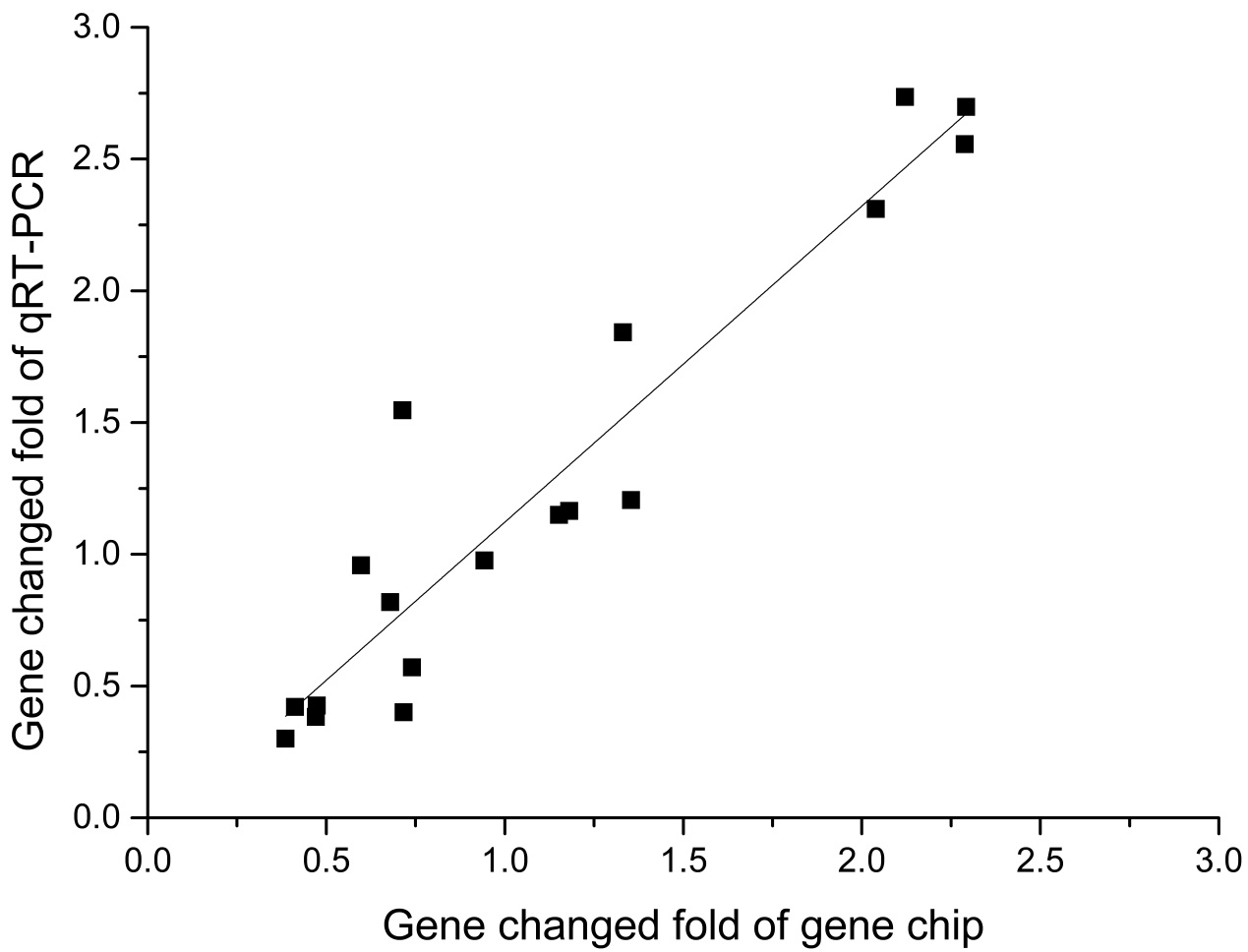


**Figure S2 |** Comparison of gene expressions determined by quantitative Real-Time PCR and DNA microarray after normalization.
